# Alveolar rhabdomyosarcoma: morphoproteomics and personalized tumor graft testing further define the biology of PAX3-FKHR(FOXO1) subtype and provide targeted therapeutic options

**DOI:** 10.18632/oncotarget.10089

**Published:** 2016-06-15

**Authors:** Robert E. Brown, Jamie Buryanek, Amanda M. Katz, Keren Paz, Johannes E. Wolff

**Affiliations:** ^1^ Department of Pathology & Laboratory Medicine, UT Health, McGovern Medical School, Houston, TX 77025, USA; ^2^ Scientific Operations, Champions Oncology, Baltimore, MD 21205, USA; ^3^ Present address: Novartis Pharmaceuticals Corporation, East Hanover, NJ 07936, USA

**Keywords:** alveolar rhabdomyosarcoma, PAX3-FKHR subtype, morphoproteomics, xenograft testing, targeted therapy

## Abstract

Alveolar rhabdomyosarcoma (ARMS) represents a block in differentiation of malignant myoblasts. Genomic events implicated in the pathogenesis of ARMS involve PAX3-FKHR (FOXO1) or PAX7-FKHR (FOXO1) translocation with corresponding fusion transcripts and fusion proteins. Commonalities in ARMS include uncontrollable proliferation and failure to differentiate. The genomic-molecular correlates contributing to the etiopathogenesis of ARMS incorporate PAX3-FKHR (FOXO1) fusion protein stimulation of the IGF-1R, c-Met and GSK3-β pathways. With sequential morphoproteomic profiling on such a case in conjunction with personalized tumor graft testing, we provide an expanded definition of the biology of PAX3-FKHR (FOXO1) ARMS that integrates genomics, proteomics and pharmacogenomics. Moreover, therapies that target the genomic and molecular biology and lead to tumoral regression and/or tumoral growth inhibition in a xenograft model of ARMS are identified.

**Significance:**

This case study could serve as a model for clinical trials using relatively low toxicity agents in both initial and maintenance therapies to induce remission and reduce the risk of recurrent disease in PAX3-FKHR (FOXO1) subtype of ARMS.

## INTRODUCTION

Alveolar rhabdomyosarcoma (ARMS) is a highly aggressive soft tissue sarcoma affecting children and adolescent age groups. At the genomic level it can be associated with translocations involving t(2;13) or t(1;13) resulting in fusion transcripts of PAX3-FKHR (FOXO1) and PAX7-FKHR (FOXO1) genes and corresponding proteins, reportedly accounting for 55% and 22% of ARMS, respectively in one study [1]. Moreover, in this same study the PAX3-FKHR group demonstrated an estimated 4-year overall survival of only 8% and with metastatic disease, a significant increased risk of failure and death. The biology of ARMS has recently been summarized to include blocks in differentiation and promotion of proliferation in the context of genomic aberrations and specifically, has incorporated the insulin-like growth factor (IGF), c-Met, GSK3-β, transforming growth factor (TGF)-beta/Gli2, YY1, and enhancer of Zeste homolog 2 (EZH2) pathways [2–17]. In short, by neutralizing the biological influence of the fusion transcript and protein, PAX3-FKHR (FOXO1) and targeting the pathways that impose both a block in differentiation and facilitate proliferation, we should be able to convert this aggressive and lethal form of ARMS to a more benign and indolent state and prevent recurrent disease.

The purpose of this report is threefold: 1. to present genomic and morphoproteomic findings in a patient with PAX3-FKHR (FOXO1) ARMS that help to further define the biology of this subtype; 2. to show, in a xenograft model, confirmation of therapies that could target its genomic and proteomic biology and the block in differentiation and lead to tumoral regression and/or tumoral growth inhibition; and 3. to propose a clinically scientific treatment regimen that could be used in both an initial and maintenance mode to target the biology of PAX3-FKHR (FOXO1) subtype of ARMS, remove the block in differentiation and minimize the risk of recurrent disease.

## RESULTS

### Patient clinical course and treatment history

A 10 year-old male had originally presented with alveolar rhabdomyosarcoma of the left hand with a positive axillary lymph node for which he underwent chemoradiation therapy (x15 months). He then recurred at age 12 with an abdominal mass in the soft tissue of the retroperitoneal region and in the subcutaneous region of the back for which he received vinorelbine, cyclophosphamide and temsirolimus, with a reported 70% tumor reduction. Unfortunately, the patient's ARMS subsequently recurred again, this time with new testicular and retroperitoneal lesions and brain metastasis for which he was treated with temozolomide and irinotecan, and then metformin and vorinostat approximately one month prior to the biopsy of the right lower quadrant mass. He died at age 14 with metastasis to the brain and leptomeningeal sarcomatosis. An autopsy also revealed extensive metastatic disease in the organs of the abdominal cavity, the retroperitoneum, diaphragm and rib cage, but with no metastatic nodules in the lung parenchyma.

### Molecular analyses

**Figure 1:**
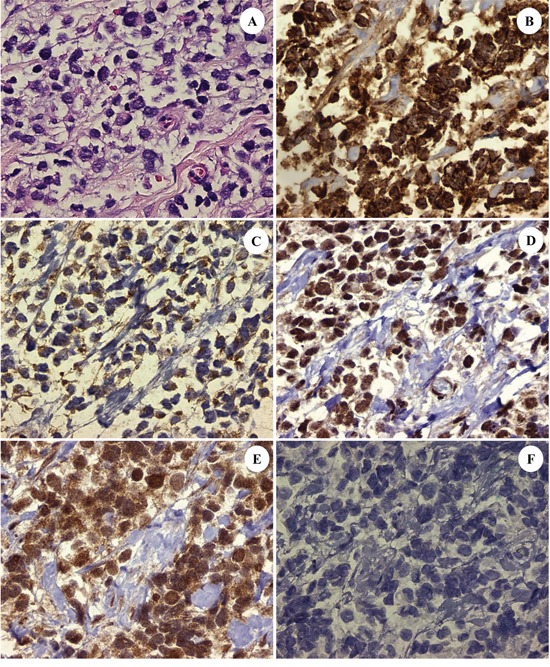
The patient's ARMS, PAX3-FKHR subtype with: H&E stained section showing dedifferentiated tumor cells **A.** high expression of insulin-like growth factor (IGF)-1 receptor [Tyr1165/1166] in the cytoplasm of the tumor cells **B.** activation of c-Met tyrosine kinase as evidenced by the moderate expression in the cytoplasm of phosphorylated (p)-c-Met (Tyr1234/1235) at up to 2+ **C.** constitutive activation of the mTORC2/Akt pathway with concomitant nuclear expression of p-mTOR (Ser 2448) and p-Akt (Ser473) consistent with mTORC2 pathway activation **D.** and **E.** and downstream signaling from IGF-1R. Contrast with negative control **F.** (DAB[3,3′-diaminobenzidine] brown chromogenic signal; original magnifications x400 for Frames A-F).

**Figure 2:**
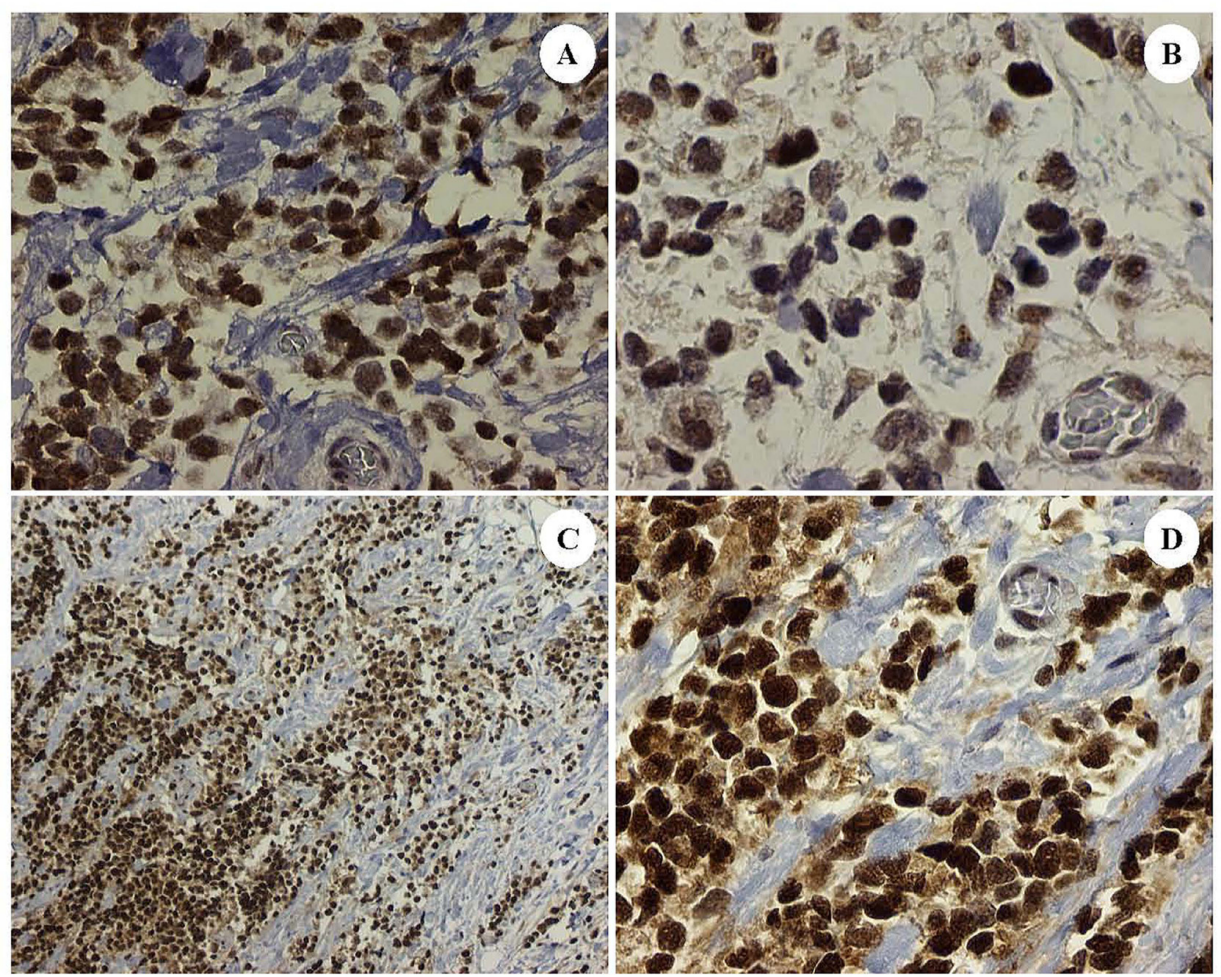
Dedifferentiated tumor cells in the patient's ARMS, PAX3-FKHR subtype showing correlative expression in tumoral nuclei of: Sirt1 **A.** Gli2 **B.** and EZH2 **C.** and **D.** DAB brown chromogenic signal, original magnifications x400 for Frames A, B and D and x100 for Frame C).

Fluorescent *in situ* hybridization (FISH) analysis was performed on a biopsy from the retroperitoneal biopsy by an outside hospital and confirmed the presence of *FKHR (FOXO1)* gene rearrangement. A subsequent independent genomic report from a different commercial laboratory confirmed the *PAX3-FKHR (FOXO1)* translocation in his ARMS. Morphoproteomic analysis [18, 19] performed on a portion of the biopsy from the abdomen demonstrated the following proteomic findings associated with correlates to the *PAX3-FKHR (FOXO1)* translocation and with its dedifferentiated state: total insulin-like growth factor −1 receptor (IGF-1R[Tyr1165/1166]) expression in the cytoplasm of virtually all of the tumor cells at 1-3+ signal intensity (on a scale of 0 to 3+)[2, 3]; constitutive activation of c-Met tyrosine kinase as evidenced by the expression of phosphorylated (p)-c-Met (Tyr1234/1235) at up to 2+[4, 5]; constitutive activation of the IGF-1R/mTORC2/Akt pathway with concomitant nuclear expression of p-mTOR (Ser 2448) and p-Akt (Ser 473) consistent with mTORC2 pathway activation and downstream signaling from the IGF-1R pathway [20–24]; Silent mating type information regulation 2 homolog 1(Sirt1) which is an NAD+ histone deacetylase and can be tumorigenic in the context of maintaining an undifferentiated primitive state and in providing active tumorigenic molecules to drive cell proliferation [25–27] was expressed at up to 3+ in the majority of the tumoral nuclei (>90%); glioma-associated oncogene protein 2 (Gli2) which can reflect signaling from the sonic hedgehog pathway and the transforming growth factor (TGF)-beta{Smad3} pathway and thereby has the potential to inhibit MyoD and contribute to a block in differentiation to a benign form [7–10] was variably expressed (0 to 3+) but in the majority of tumoral nuclei; and Enhancer of Zeste homolog 2 (EZH2), a histone methyltransferase was immunopositive in the majority of the tumoral nuclei consistent with both the repression of genes that are associated with stem cell differentiation and collaboration with JARID2, a downstream target of PAX3-FKHR leading to the inhibition of myogenic differentiation [12–17]. The digital images from the morphoproteomic analysis along with the microanatomical features of the tumor are depicted in Figures 1 and 2. Further consideration of the genomic and proteomic correlates and their application to targeted therapy are contained below in the Discussion.

### Xenograft testing

**Figure 3:**
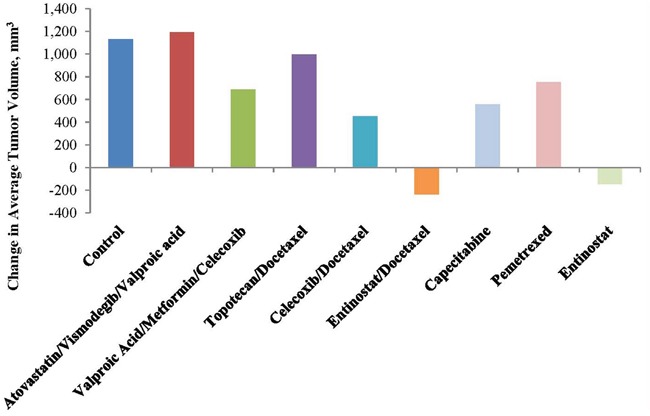
Relative growth of treatment groups at test completion Note tumor regression with entinostat and tumor growth inhibition with valproic acid, a class I histone deacetylase inhibitor, celecoxib and metformin.

**Figure 4:**
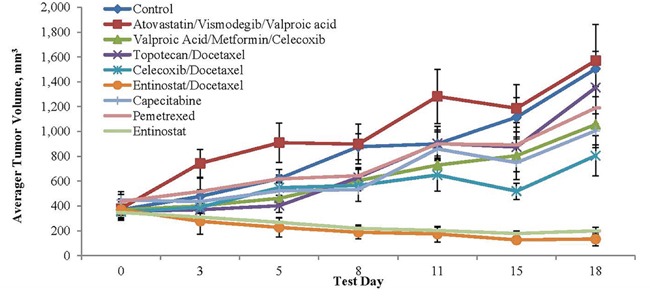
Tumor volume and agent activity data plotted against test days 0 to 18 Note tumor regression over time with entinostat and tumor growth inhibition over time with valproic acid, a class I histone deacetylase inhibitor, celecoxib and metformin vis-à-vis the control.

A personalized model of the antitumor activity of selected therapies was created from a portion of the patient's ARMS, also obtained via biopsy of the recurrent tumor from the right lower quadrant of his abdomen (Personalized Champions TumorGraft^®^ Test). The combinatorial or individual therapies that effected either tumor regression (TR) and/or tumor growth inhibition (TGI) included the following: TR following treatment with Entinostat alone or combined with docetaxel (Figure 3 and Table 1); and TGI seen with valproic acid+ metformin +celecoxib (39%), with celecoxib +docetaxel (60%), with entinostat + docetaxel (121%), and with entinostat alone (113%) [Figure 4 and Table 1]. These data only became available after the patient was deceased.

**Table 1:** Tumor volume and agent activity(%TGI=tumor growth inhibition;%TR=tumor regression)

Group	% TGI	RECIST PD/SD/PR/CR*	%TR
**Control**		6/0/0/0	
Atovastatin Vismodegib Valproic Acid	n/a	3/0/0/0	n/a
Valproic Acid Metformin Celecoxib	39	3/0/0/0	n/a
Topotecan Docetaxel	12	3/0/0/0	n/a
Celecoxib Docetaxel	60	3/0/0/0	n/a
Entinostat Docetaxel	121	0/0/3/0	65
Capecitabine	51	3/0/0/0	n/a
Pemetrexed	34	3/0/0/0	n/a
Entinostat	113	0/1/3/0	43

PD-Progressive Disease; SD-Stable Disease; PR-Partial Response; CR-Complete Response

## DISCUSSION

Genomic testing of this patient's ARMS confirmed it as *PAX3-FKHR (FOXO1)* subtype. Furthermore, the aggressive nature of this tumor and the unfortunate eventual outcome coincides with the behavior and high mortality of this subtype. Using morphoproteomic analysis in conjunction with the genomic characterization of the patient's tumor as *PAX3-FKHR (FOXO1)* subtype and data from xenograft testing of his tumor in the context of data mining of the National Library of Medicine's MEDLINE data base, we have further characterized the biology of this subtype and provide genomic, proteomic and pharmacogenomics correlates to targeted therapies.

**Figure 5:**
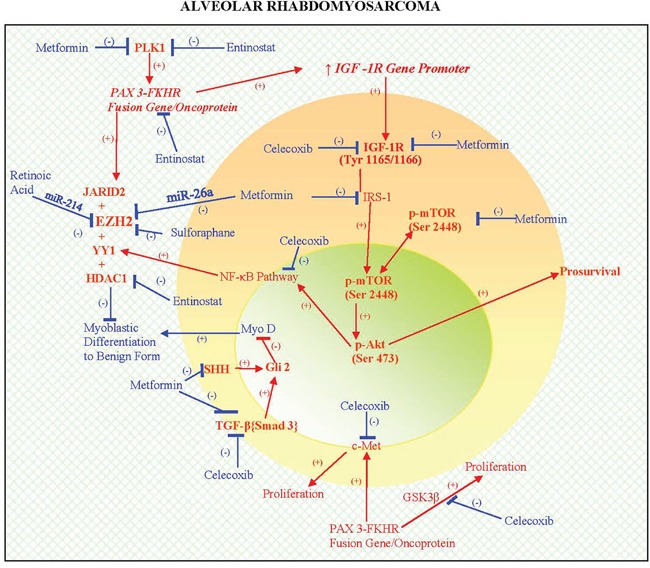
A schematic depicting the biology of PAX3-FKHR (FOXO1) subtype of ARMS that integrates genomics and proteomics in a pathogenetic sequence, resulting in a dedifferentiated state and promoting proliferation (see 
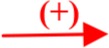
) Moreover, therapies that can target the genomics and molecular biology and take advantage of pharmacogenomics to relieve the block in differentiation to a more benign form and result in reduced proliferation are also illustrated in the schematic (see 
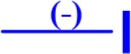
) and covered in detail in the Discussion. A proof of concept is provided in part by the results of xenograft testing of the patient's ARMS, PAX3-FKHR (FOXO1) subtype with the application of entinostat showing tumor regression and a combination of valproic acid, a class I histone deacetylase inhibitor, celecoxib and metformin showing tumor growth inhibition (see Figures 3 and 4, Table 1 and Discussion).

PAX3-FKHR (FOXO1) fusion gene/protein correlates with the following morphoproteomic findings in this patient's ARMS: high expression levels of the protein analyte, total IGF-1R (Tyr1165/1166) consistent with the ability of the fusion oncoprotein to upregulate the transcription of the *IGF-1receptor* gene promoter resulting in the corresponding protein [2, 3]; and expression with constitutive activation of c-Met (phosphorylated on tyrosine 1234/1235) consistent with the role of the fusion protein in upregulating MET and inducing a ligand-independent activation of Met signaling, a proproliferative pathway in ARMS [4, 5]. Additional downstream signaling of PAX3-FKHR (FOXO1) fusion gene/oncoprotein include the proproliferative GSK3beta pathway [6] and JARID2, the latter enabling the collaboration of EZH2 with YY1. Parenthetically, YY1 in this collaboration with JARID2 can recruit EZH2 and HDAC 1 to the promoter region leading to a block in differentiation, partially via the downregulation of miR-29b2 and miR-29c and by inhibiting MyoD, favoring rhabdomyosarcoma cell proliferation over differentiation [11–17, 28]. Phosphorylative activation of PAX3-FOX01 by polo-like kinase (PLK) 1 [29] is noteworthy as a potential contributor to the pathogenesis of this subtype of ARMS. Moreover, the correlative expression of the IGF-1R/mTORC2/Akt pathway with constitutive activation of p-Akt (Ser 473) in his tumor could have played a dual role in ARMS. By activating the downstream nuclear factor (NF)-kappaB pathway [18, 19], p-Akt could facilitate the availability of YY1 and thereby the block in differentiation and conversely, p-Akt (Ser 473) can exert feedback inhibition of PAX3-FKHR [30]. The latter coincides with the clinical response in the patient during temsirolimus therapy given that rapalogs can initially upregulate the IGF-1R/mTORC2/Akt pathway [24, 31] and could have temporarily moderated the PAX3-FKHR signaling. Finally, other factors that are likely to have imposed a block in myoblastic differentiation include Sirt1 and Gli2, which were detected by morphoproteomic analysis. These pathogenetic factors of this PAX3-FKHR (FOXO1) subtype of ARMS imposing a block in differentiation and favoring proliferation are depicted in the schematic (see Figure 5).

In this context, the following agents should act to address the biology of ARMS by acting at genomic and proteomic levels: 1) Entinostat by virtue of its ability to: a) inhibit class I HDAC [32] and thereby, to interrupt the YY1-EZH2-HDAC1 inhibition of miR-29b2 and mir-29c allowing arrest of ARMS cell proliferation and promoting differentiation [11–17, 28]; and b) its ability to effect direct transcriptional supression of PAX3:FOXO1 [33] and to inhibit the phosphorylative activation of PAX3—FOXO1 by polo-like kinase (PLK)1 [29, 34, 35]; 2) celecoxib inhibits the NF-kappaB pathway at multiple points [36] and should reduce the overproduction of YY1; 3) sulforaphane, a nutraceutical suppresses polycomb group protein level including EZH2 [37] and this should promote alveolar rhabdomyosarcoma's differentiation to a more benign form, induce apoptosis in the tumor cells [38] and reduce the survival of alveolar rhabdomyosarcoma leading to elimination of the tumor (in the latter study, sulforaphane was also shown to decrease the mRNA and protein levels of PAX3-FKHR, MYCN and MET in ARMS cells); 4) retinoic acid upregulates miR-214 which downregulates EZH2 protein in embryonic stem cells and miR-214-mediated EZH2 protein reduction accelerates skeletal muscle cell differentiation [39] (Both all-trans retinoic acid, ATRA and 9-cis retinoic acid suppressed the cell growth of alveolar rhabdomyosarcoma with evidence of a differentiating effect [40]. ATRA increased the expression of some genes associated with muscle differentiation and slowed the proliferation and promoted a more differentiated myogenic phenotype in ARMS cell lines [41]. A phase I study with Entinostat, in combination with 13-cis-retinoic acid for patients with solid tumors but none with ARMS was completed and showed that these were reasonably well tolerated in the dosing regimen used [42]); and 5) metformin should be effective by virtue of its ability to: a) upregulate miR-26a and thereby downregulate EZH2 [43] (miR-26a has been shown to be low and EZH2mRNA high in ARMS cells and patient samples vis-à-vis controls [44]); b) its ability to reduce the signaling to the NF-kappaB pathway by virtue of its ability to inhibit the IGF-1R/mTORC2/Akt pathway [18, 19, 24]; c) its ability to suppress polo-like kinase genes via activation of AMPK [45] and to interfere in the TGF-beta{Smad}3/Gli2 pathway (*vide supra* [7–10, 46–48]). These pathways and therapeutic agents are schematically represented in the diagram below (see Figure 5). Proofs of concept are also provided by the results of xenograft testing of the patient's PAX3-FKRH (FOXO1) subtype of ARMS (*vide supra*). Specifically, tumor regression and tumor growth inhibition were effected with Entinostat alone and tumor growth inhibition was achieved with a combination of Valproic Acid, a class I histone deacetylase inhibitor, celecoxib and metformin.

In summary, we present genomic and morphoproteomic findings in a patient with PAX3-FKHR (FOXO1) subtype of ARMS that further define the existing biology of this subtype of ARMS and correlate these with a block in myoblastic differentiation of this malignant process to a more benign form. Using data mining of the National Library of Medicine's MEDLINE database, we identified therapeutic targets and agents acting in pharmacoproteomic and pharmacogenomic fashion that could remove the etiopathogenetic block to myoblastic differentiation in this subtype. Proofs of concept are also provided from xenograft testing of this tumor using agents that interrupt the pathways that block differentiation and also effected tumor regression and/or tumor growth inhibition. These are provided as a basis for clinical scientific consideration as we build a therapeutic strategy to target the PAX3-FKHR (FOXO1) subtype of ARMS. To that end, we contemplate offering a pilot trial for patients with the PAX3-FKHR (FOXO1) subtype who have recurrent disease. Such a trial will incorporate morphoproteomic-guided targeted therapies designed to remove blocks in myoblastic differentiation to a more benign form, and will be applied in a maintenance mode with a low toxicity profile in an effort to reduce the risk of recurrent disease.

## MATERIALS AND METHODS

With Institutional Review Board approval to perform follow-up on patients on whom morphoproteomic analysis had been performed (IRB#HSC-MS-11-0410), we reviewed the medical records and test results of a patient with ARMS, who had been referred to the CONSULTATIVE PROTEOMICS service at UTHealth-Medical School at Houston for morphoproteomic analysis. Xenograft testing was performed by Champions Oncology Inc.

### Morphoproteomics

The application of bright-field microscopy and immunohistochemistry directed against protein analytes can contribute to the defining of the biology of a disease process and facilitate the integration of genomic, proteomic and pharmacogenomics events into its etiopathogenesis and provide rationale for targeted therapies. To that end, we applied immunohistochemical probes against the following protein analytes to formalin-fixed paraffin-embedded sections of the patient's recurrent ARMS in the right lower quadrant: total insulin-like growth factor −1 receptor (IGF-1R[Tyr1165/1166] (Gen Way Biotech Inc. San Diego, CA); c-Met, phosphorylated on tyrosine 1234/1235 (Cell Signaling Technology, Inc, Danvers, MA); mammalian target of rapamycin (mTOR), phosphorylated on serine 2448 (Cell Signaling Technology, Inc); Akt, phosphorylated on serine 473 (Cell Signaling Technology, Inc); silent mating type information regulation 2 homolog 1 (Sirt1)(Abcam Inc. [E104;ab32441]); glioma-associated oncogene protein 2 (Gli2)(Abcam, Inc[ab26056]); and enhancer of Zeste homolog 2 (EZH2, Cell Signaling Technology, Inc). The details of the morphoproteomic staining procedure have been previously described [49].

### Xenograft testing

An explant from the patient's right lower quadrant recurrent ARMS was referred to Personalized Champions Tumorgraft^®^ chemosensitivity testing and immediately implanted into immunodeficient mice. Per Champions Oncology, the following details are stated verbatim for the materials and methodologies employed:

#### Agent efficacy

All test agents were formulated according to manufacturer's specifications. Beginning Day 0, tumor dimensions were measured twice weekly by digital caliper and data, including individual and mean estimated tumor volumes (Mean TV ± SEM), are recorded for each group. Tumor volume was calculated using the formula: *TV= width^2^ x length x π/6*.

#### Tumor growth inhibition and RECIST

At study completion, percent tumor growth inhibition (%TGI) values were calculated and reported for each treatment group (T) versus control (C) using initial (i) and final (f) tumor measurements by the formula: *%TGI=[1-(Tf-Ti)/(Cf-Ci)]x100*. Individual mice reporting a tumor volume >120% of the Day 0 measurement are considered to have progressive disease (PD). Individual mice with neither sufficient shrinkage nor sufficient tumor volume increases are considered to have stable disease (SD). Individual mice reporting a tumor volume ≤70% of the Day 0 measurement for two consecutive measurements over a seven day period are considered partial responders (PR). If the PR persisted until study completion, percent tumor regression (%TR) is determined using the formula: *%TR=(1-Tf/Ti)x100*; a mean value is calculated for the entire treatment group. Individual mice lacking palpable tumors for two consecutive measurements over a seven day period are classified as complete responders (CR). All data collected in this study was managed electronically and stored on a redundant server system. The clinical specificity for this test is 60%; the clinical sensitivity of this test is 98.1%.
